# Gargling for Oral Hygiene and the Development of Fever in Childhood: A Population Study in Japan

**DOI:** 10.2188/jea.JE20100181

**Published:** 2012-01-05

**Authors:** Tatsuya Noda, Toshiyuki Ojima, Shinya Hayasaka, Chiyoe Murata, Akihito Hagihara

**Affiliations:** 1 Department of Community Health and Preventive Medicine, Hamamatsu University School of Medicine, Hamamatsu, Japan; 2 Department of Health Care Administration and Management, Kyushu University Graduate School of Medicine, Fukuoka, Japan

**Keywords:** gargling, fever onset, sickness absence, green tea, child health

## Abstract

**Background:**

Fever is one of the most common symptoms among children and is usually caused by respiratory infections. Although Japanese health authorities have long recommended gargling to prevent respiratory infections, its effectiveness among children is not clear.

**Methods:**

The children in this observational study were enrolled from 145 nursery schools in Fukuoka City, Japan. Children in the exposure group were instructed to gargle at least once a day. The endpoints of this study were incidence of fever during the daytime and incidence of sickness absence. Differences among gargling agents for each endpoint were also analyzed.

**Results:**

A total of 19 595 children aged 2 to 6 years were observed for 20 days (391 900 person-days). In multivariate logistic regression, the overall odds ratio (OR) for fever onset in the gargling group was significantly lower (OR = 0.68). In age-stratified analysis, ORs were significantly lower at age 2 (OR = 0.67), 4 (OR = 0.46), and 5 (OR = 0.41) years. Regarding sickness absence, the overall OR was 0.92 (not significant) in the gargling group. In age-stratified analysis, ORs were significantly lower at age 4 (OR = 0.68), 5 (OR = 0.59), and 6 (OR = 0.63) years. In subgroup analysis, significantly lower ORs for fever onset were observed for children who gargled with green tea (OR = 0.32), functional water (OR = 0.46), or tap water (OR = 0.70). However, the ORs were not significant for sickness absence.

**Conclusions:**

Gargling might be effective in preventing febrile diseases in children.

## INTRODUCTION

Fever is one of the most common symptoms of disease in childhood and results in psychological and economic burdens for patients and their families. The prevention of febrile diseases therefore plays an important role in child health. The most common cause of fever in childhood is respiratory infection.^1^ However, the evidence for putative preventive approaches for such diseases in childhood is not yet conclusive.

The custom of gargling as a preventive approach is not widespread in many Western countries. In Japan, however, health authorities have officially recommended gargling to prevent respiratory infections for more than 90 years, and almost all Japanese believe in the preventive effect of gargling.^2^^,^^3^ Although the effectiveness of gargling had long been unproven, a recent randomized controlled study in Japan showed that gargling with tap water inhibited the onset of upper respiratory tract infections among adults.^4^ Another study suggested that gargling among adults had beneficial economic effects.^5^ Nevertheless, the effectiveness of gargling among children remains to be clarified.

As an initial step in collecting the necessary data, we conducted an observational survey of a large number of children. Because it would have been prohibitively expensive to investigate complicated outcomes requiring diagnosis by a doctor, we focused on overall febrile disease as a proxy of respiratory infections among children. Our aim in this large-scale population survey was to examine whether gargling prevented development of fever and the incidence of sick absence among children.

## METHODS

### Subjects and protocol

The Fukuoka Preschool Health Study was conducted by the Joint Committee for Preschool Children’s Health of the Fukuoka City Medical Association from January through February 2006. The eligible facilities were all 166 mayor-authorized nursery schools in Fukuoka City, Japan, of which 145 agreed to join this study.

We conducted a follow-up study of cohorts identified in the Fukuoka Preschool Health Study. The observation period was 20 weekdays between January and February 2006. The inclusion criterion was attendance at any of the participating schools. We excluded children who: were too young to gargle (ie, younger than 2 years), had disabilities that are associated with febrile diseases or hindrance of gargling, withdrew from the study, and did not answer all required questions. First, children younger than 2 years were excluded. Then, children who met the other exclusion criteria were excluded.

The baseline characteristics and health status information of the children were collected by using a teacher-administered questionnaire, which asked about sex, age, body temperature (if feverish), dates of absence, reasons for absence (if absent), size and location of school, and whether they gargled once or more a day at nursery school. Some classrooms in each school had a policy of letting children gargle; others did not. Although gargling information was collected by a questionnaire and analyzed on an intention-to-treat basis, a classroom teacher instructed children to gargle at all scheduled times and visually confirmed that they had gargled. Gargling was conducted by rinsing the throat (*garagara-ugai* method) at least once a day with any of the following agents: tap water, saline water, green tea, and functional water (alkali ion water or ozone water). Body temperature was measured by the classroom teacher, and fever onset was defined as a body temperature of 37.5°C (99.5°F) or higher. Each teacher recorded dates of absence and, when a child was absent, the reason for absence was obtained from the children’s parents.

### Statistical analyses

One-way analysis of variance was used for group comparisons of numerical variables, and the chi-square test was used for comparisons of categorical data. We then used logistic regression analyses for repeated measurements for correlations between repeated measures of individuals, using generalized estimating equations.^6^ The primary study endpoint was incidence of fever during the daytime. We used incidence of sickness absence as a surrogate endpoint because nursery school regulations recommended that children with a high fever at night should not attend school the next day. In addition, the differences among gargling agents for each endpoint was tested using logistic regression for repeated measurement. All statistical analyses were performed with SAS (version 9.1).

### Ethical approval

All participating nursery schools provided written informed consent for the acquisition and research use of data. The governing board of the Fukuoka City Medical Association granted ethical approval, and we obtained anonymized datasets before beginning the study analyses.

## RESULTS

From among 22 692 children recruited at 145 nursery schools, 2528 who were younger than 2 years were excluded, after which an additional 569 children were excluded because they met other exclusion criteria. Ultimately, 19 595 children aged 2 to 6 years were observed for 20 days (391 900 person-days).

Table 1 shows the baseline characteristics of the participants. The size of schools was categorized according to quartiles of the number of children in the school, and school location in Fukuoka City was classified by city ward. No significant differences were observed between sexes. There were significant differences between the gargling and non-gargling groups in the distribution of age, school size, and school location.

**Table 1.** Baseline characteristics of participants by gargling status

	Gargling	Non-gargling	
(Total: *n* = 19 595)	(*n* = 15 859)	(*n* = 3736)	*P* value
Age			
mean age (± SD)	4.48 (± 1.16)	2.42 (± 0.71)	<0.001
Sex			
male	8207 (80.8%)	1945 (19.2%)	0.126
female	7652 (81.0%)	1791 (19.0%)	
Size of school (number of children)			
<139	3931 (80.6%)	946 (19.4%)	<0.001
139–168	3930 (78.4%)	1086 (21.7%)	
169–224	3800 (78.5%)	1042 (21.5%)	
225–358	4198 (86.4%)	662 (13.6%)	
Location of school (ward)			
Higashi	3844 (80.2%)	951 (19.8%)	<0.001
Sawara	2629 (80.9%)	621 (19.1%)	
Minami	2407 (82.0%)	530 (18.1%)	
Hakata	2445 (83.9%)	468 (16.1%)	
Nishi	2157 (79.8%)	545 (20.2%)	
Jonan	1373 (76.4%)	425 (23.6%)	
Chuo	1062 (83.8%)	206 (16.3%)	

Data are numbers unless otherwise indicated.

The rates for fever onset and sickness absence were significantly lower in the gargling group than in the non-gargling group (Table 2). Although the proportion of gargling children across the 145 schools ranged from 18.2% to 100% (interquartile range: 70.4%–88.8%), the proportion across the 7 wards ranged from 76.3% to 83.9%. The rates of fever outcomes across each size of school ranged from 0.28% to 0.40% in the gargling group and from 0.85% to 1.31% in the non-gargling group. Furthermore, the rates of fever outcomes across 7 wards ranged from 0.21% to 0.49% in the gargling group and from 0.59% to 1.46% in the non-gargling group. We divided all schools into 4 quartiles, based on the proportions of gargling children in attendance, to compare the rates of fever outcomes in non-gargling groups among the 4 school groups. There was no trend in the rate of fever in the non-gargling group over the 4 school groups.

**Table 2.** Comparison of outcomes in gargling and non-gargling children

	Gargling	Non-gargling	
(Total: 391 900 person-days)	(317 180 person-days)	(74 720 person-days)	*P* value
Fever onset			
Yes	1095 (0.4%)	711 (1.0%)	<0.001
No	316 085 (99.7%)	74 009 (99.1%)	
Sickness absence			
Yes	12 672 (4.0%)	3874 (5.2%)	<0.001
No	304 508 (96.0%)	70 846 (94.8%)	

Data are numbers unless otherwise indicated.

The results of logistic regression are shown in Tables 3 and 4. Each table shows the 4 models used to estimate the odds ratio for gargling. We first conducted the analyses adjusted by age (models 1 and 2). However, considering the strong influence of age on outcomes, analyses stratified by age were added (models 3 and 4). As shown in Table 3, gargling was associated with significantly lower odds ratios for fever onset, except among children aged 3 (in model 4) and 6 years. The odds ratios for sickness absence were significantly lower among gargling children aged 4 or 5 years and among those aged 6 years in model 3 (Table 4).

**Table 3.** Fever onset and gargling status in children aged 2–6 years

(Adjusted by age)	Model 1	Model 2
	
Gargling *v.* non-gargling	0.72 (0.61–0.86)	0.68 (0.57–0.82)

(Stratified by age)	Model 3	Model 4
	
Age 2 (*n* = 3251; *g* = 742, *ng* = 2509)
Gargling *v.* non-gargling	0.71 (0.56–0.91)	0.67 (0.53–0.86)
Age 3 (*n* = 3675; *g* = 2681, *ng* = 994)
Gargling *v.* non-gargling	0.81 (0.61–0.92)	0.75 (0.52–1.08)
Age 4 (*n* = 4424; *g* = 4271, *ng* = 153)
Gargling *v.* non-gargling	0.40 (0.24–0.68)	0.46 (0.26–0.80)
Age 5 (*n* = 4521; *g* = 4474, *ng* = 47)
Gargling *v.* non-gargling	0.39 (0.18–0.84)	0.41 (0.18–0.93)
Age 6 (*n* = 3724; *g* = 3691, *ng* = 33)
Gargling *v.* non-gargling	N/C	N/C

Data are odds ratios (95% confidence interval)

*n*: total number of children, *g*: number of gargling children, *ng*: number of non-gargling children

Reference: non-gargling, N/C: regression model did not converge

Model 1: gargling status and age; Model 2: gargling status, sex, age, school location, and school size

Model 3: gargling status; Model 4: gargling status, sex, school location, and school size

**Table 4.** Sickness absences and gargling status in children aged 2–6 years

(Adjusted by age)	Model 1	Model 2
	
Gargling *v.* non-gargling	0.95 (0.87–1.05)	0.92 (0.84–1.00)

(Stratified by age)	Model 3	Model 4
	
Age 2 (*n* = 3251; *g* = 742, *ng* = 2509)
Gargling *v.* non-gargling	1.04 (0.90–1.20)	0.97 (0.85–1.10)
Age 3 (*n* = 3675; *g* = 2681, *ng* = 994)
Gargling *v.* non-gargling	1.02 (0.88–1.17)	0.96 (0.83–1.10)
Age 4 (*n* = 4424; *g* = 4271, *ng* = 153)
Gargling *v.* non-gargling	0.65 (0.51–0.84)	0.68 (0.52–0.87)
Age 5 (*n* = 4521; *g* = 4474, *ng* = 47)
Gargling *v.* non-gargling	0.57 (0.37–0.88)	0.59 (0.38–0.92)
Age 6 (*n* = 3724; *g* = 3691, *ng* = 33)
Gargling *v.* non-gargling	0.61 (0.38–0.99)	0.63 (0.38–1.04)

Data are odds ratios (95% confidence interval)

*n*: total number of children, *g*: number of gargling children, *ng*: number of non-gargling children

Reference: non-gargling

Model 1: gargling status and age; Model 2: gargling status, sex, age, school location, and school size

Model 3: gargling status; Model 4: gargling status, sex, school location, and school size

The gargling agents were added to the model in subgroup analysis (Table 5). As compared with the non-gargling group, each agent was associated with significantly lowered odds ratios for the incidence of fever onset, except for saline water in model 2. In particular, gargling with green tea yielded an odds ratio of approximately 0.3. In contrast, no gargling agent had a clear effect on sickness absences.

**Table 5.** Gargling agents and fever onset/sickness absences in children aged 2–6 years

		Model 1	Model 2
**Fever onset**			
Non-gargling^a^	(*n* = 3736)	1.00	1.00
Tap water	(*n* = 14 190)	0.74 (0.62–0.88)	0.70 (0.58–0.85)
Saline water	(*n* = 173)	0.40 (0.18–0.90)	0.50 (0.22–1.12)
Green tea	(*n* = 407)	0.29 (0.16–0.55)	0.32 (0.17–0.61)
Functional ​ water	(*n* = 306)	0.50 (0.27–0.93)	0.46 (0.24–0.86)
**Sickness absence**			
Non-gargling^a^	(*n* = 3736)	1.00	1.00
Tap water	(*n* = 14 190)	0.94 (0.86–1.04)	0.94 (0.86–1.04)
Saline water	(*n* = 173)	1.17 (0.83–1.64)	1.12 (0.80–1.59)
Green tea	(*n* = 407)	1.15 (0.93–1.44)	1.12 (0.90–1.40)
Functional ​ water	(*n* = 306)	0.96 (0.77–1.21)	0.98 (0.77–1.25)

Data are odds ratios (95% confidence interval)

Model 1: Adjusted for gargling agent and age

Model 2: Adjusted for gargling agent, age, school location, and school size

^a^Reference

## DISCUSSION

To our knowledge, this is the first study to assess the effectiveness of gargling in preventing febrile diseases and sickness absences among healthy children. We found that gargling once or more a day was associated with lower onset of both febrile diseases during the daytime in children aged 2 to 6 years and sickness absences in children aged 4 to 6. The effect on prevention of febrile diseases differed by gargling agent.

In a large proportion of children, fever is caused by viral respiratory infections (eg, rhinovirus, coronavirus, RS virus, influenza virus, and adenovirus), followed by bacterial respiratory infections (eg, streptococci, pneumococci, and Moraxella catarrhalis).^7^^–^^9^ Other febrile diseases (eg, urinary tract infections, otitis media, etc.) account for a small percentage or less than 1% of febrile disease in children.^10^

The results of this study and the site of action of gargling suggest that gargling might prevent viral upper respiratory tract infections. However, the incubation times of the viruses mentioned above are less than 12 hours, and the viruses bind to specific cell receptors.^11^^–^^13^ Thus, it is uncertain whether simple flushing could actually wash out the viruses. In 2 previous studies showing that gargling prevented adult upper respiratory tract infections (mainly induced by viral infections), the authors also could not explain how gargling prevented viral infection.^4^^,^^14^ A plausible explanation is that chlorine is added to tap water by law. The concentration of residual chlorine in tap water in Fukuoka City was 0.60 mg/L at the time of our study, which would have ensured inactivation of viruses and bacteria.^15^^,^^16^ Another explanation was suggested by a randomized clinical trial showing that oral rinsing with chlorhexidine gluconate for 30 seconds twice a day reduced the total nosocomial respiratory infection rate among patients in a cardiovascular intensive care unit.^17^ The authors of that article noted that reducing oropharyngeal microbial flora by oral rinsing with an antiseptic agent twice a day could have impeded aspiration of organisms that cause respiratory infections. Although the above hypotheses are consistent with our findings, more laboratory research is needed to clarify the mechanism by which gargling prevents disease.

Gargling was associated with a lower incidence of sickness absences in several age groups; however, the odds ratios were close to unity, possibly because some sickness absences were caused by diseases or symptoms other than respiratory diseases, such as enteritis and otitis media. To better understand the effect of nocturnal fever onset, the reasons for absences associated with fever should be selectively extracted and analyzed. However, the reasons reported by parents varied widely and were often too vague to identify a specific disease (eg, “hoarseness,” “looking sick,” or “not doing well”). Therefore, we could not confirm the effect of gargling on sickness absences or determine why the odds ratio for nocturnal fever onset was close to unity in gargling children.

Regarding gargling agents, we found that gargling with green tea had a greater impact on febrile disease. The results of a prospective cohort study of the effect of gargling with green tea suggested that such gargling lowers the risk of influenza infection, although the effect was not significant in that study.^18^ Tea catechin is a type of flavonoid in green tea and has antiviral and bacteriocidal effects.^19^^–^^22^ In a previous clinical study, gargling with a catechin extract of green tea inhibited influenza infection, and application of green tea extract to the oral or nasal cavities suppressed various pathogenic bacteria.^23^^,^^24^ The effect of green tea in our study might thus be related to tea catechin.

Previous studies of gargling were relatively small. The main advantage of this study is that it was a large-scale study of approximately 20 000 children, which ensured high statistical power. The preventive effect of gargling is affected by the prevalence of the target disease; however, there was no mass outbreak of influenza or other major febrile infection in Fukuoka City during the study period, according to the Fukuoka City health authorities.

There were 2 main limitations in this study. First, because this was an observational study, assignment was not randomized or masked to the subjects. Randomization is preferable, as it avoids potential confounding factors. However, randomization of gargling is difficult, as it is believed to be a beneficial behavior. To minimize the effect of potential confounding factors, we conducted analyses stratified by age, which was considered a major confounding factor, and included the location of the school (ward) in some models. Since there was some variability among wards in the proportion of fever onset in the gargling group, the location of the school was assumed to be a confounding factor in the effect of gargling. We postulated that differences in socioeconomic status (SES) played a major role in this variability. SES is believed to be associated with susceptibility to respiratory infection, and previous studies suggested that decreases in SES were correlated with lower susceptibility.^25^ Unfortunately, data on parameters that directly reflect SES were not collected in this study, and we could not acquire information on the SES of each school or child after the survey. The effect of SES, therefore, was not fully adjusted and should be addressed in future surveys. Second, we did not collect detailed information on the method of gargling and other practices of infection prevention (especially hand washing). However, it is unlikely that they varied greatly among children in Japan because gargling and hand-washing are widely practiced throughout the country, and children receive instructions on gargling and hand-washing technique. In addition, both the child health authority of Fukuoka City and Fukuoka Medical Association provided explicit instruction to participating nurseries to encourage children to wash their hands.

It is important for preschool children to perform infection control techniques in nursery school because infectious diseases tend to be spread in such environments. Gargling is an inexpensive and straightforward hygienic measure for children and can be done anywhere. Green tea is a common and safe beverage in Japan, and there seem to be few problems in using it as a gargling agent. While gargling is not a popular decontamination method, except in some Asian countries, there is no reason to hesitate to use it as a preventive measure against febrile diseases in childhood.

## References

[1] Robert MK, Karen M, Hal BJ, Richard EB. Nelson Essentials of Pediatrics. 5th ed. Philadelphia: Elsevier Saunders; 2006. p. 461.

[2] ABC’s of the history of influenza pandemic. The Yomiuri Shimbun. 2009 Jun 4 (in Japanese).

[3] Ministry of Health, Labour and Welfare. Guideline for new type of influenza management. Tokyo: Ministry of Health, Labour and Welfare; 2007.

[4] Satomura K, Kitamura T, Kawamura T, Shimbo T, Watanabe M, Kamei M, Prevention of upper respiratory tract infections by gargling: a randomized trial. Am J Prev Med. 2005;29:302–7 10.1016/j.amepre.2005.06.013 16242593

[5] Sakai M, Shimbo T, Omata K, Takahashi Y, Satomura K, Kitamura T, Cost-effectiveness of gargling for the prevention of upper respiratory tract infections. BMC Health Serv Res. 2008;8:258 10.1186/1472-6963-8-258 19087312 PMC2651874

[6] Liang KY, Zeger SL Longitudinal data analysis using generalized linear models. Biometrika. 1986;73:13–22 10.1093/biomet/73.1.13

[7] Baraff LJ Management of fever without source in infants and children. Ann Emerg Med. 2000;36:602–14 10.1067/mem.2000.110820 11097701

[8] Lopez JA, McMillin KJ, Tobias-Merrill EA, Chop WM Jr Managing fever in infants and toddlers: toward a standard of care. Postgrad Med. 1997;101:241–2, 245–52 10.3810/pgm.1997.02.168 9046938

[9] Moyer V, Elliott E. Evidence Based Pediatrics and Child Health. 1st ed. London: BMJ Books; 2000. p. 169–77.

[10] Browne GJ, Currow K, Rainbow J Practical approach to the febrile child in the emergency department. Emerg Med (Fremantle). 2001;13:426–35 10.1046/j.1035-6851.2001.00256.x 11903427

[11] Gwaltney JM Jr Clinical significance and pathogenesis of viral respiratory infections. Am J Med. 2002;112 Suppl 6A:13S–8S 10.1016/S0002-9343(01)01059-2 11955455

[12] Bella J, Rossmann MG Review: rhinoviruses and their ICAM receptors. J Struct Biol. 1999;128:69–74 10.1006/jsbi.1999.4143 10600561

[13] Reithmayer M, Reischl A, Snyers L, Blaas D Species-specific receptor recognition by a minor-group human rhinovirus (HRV): HRV serotype 1A distinguishes between the murine and the human low-density lipoprotein receptor. J Virol. 2002;76:6957–65 10.1128/JVI.76.14.6957-6965.2002 12072496 PMC136340

[14] Kitamura T, Satomura K, Kawamura T, Yamada S, Takashima K, Suganuma N, Can we prevent influenza-like illnesses by gargling? Intern Med. 2007;46:1623–4 10.2169/internalmedicine.46.0104 17878657

[15] Shirai J, Kanno T, Tsuchiya Y, Mitsubayashi S, Seki R Effects of chlorine, iodine, and quaternary ammonium compound disinfectants on several exotic disease viruses. J Vet Med Sci. 2000;62:85–92 10.1292/jvms.62.85 10676896

[16] Thurston-Enriquez JA, Haas CN, Jacangelo J, Gerba CP Chlorine inactivation of adenovirus type 40 and feline calicivirus. Appl Environ Microbiol. 2003;69:3979–85 10.1128/AEM.69.7.3979-3985.2003 12839771 PMC165174

[17] DeRiso AJ 2nd, Ladowski JS, Dillon TA, Justice JW, Peterson AC Chlorhexidine gluconate 0.12% oral rinse reduces the incidence of total nosocomial respiratory infection and nonprophylactic systemic antibiotic use in patients undergoing heart surgery. Chest. 1996;109:1556–61 10.1378/chest.109.6.1556 8769511

[18] Yamada H A randomized controlled study on the effects of gargling with tea catechin extracts on the prevention of influenza infection in healthy adults. Jpn J Clin Pharmacol Ther. 2007;38:323–30 10.3999/jscpt.38.323

[19] Yamada H, Ohashi K, Atsumi T, Okabe H, Shimizu T, Nishio S, Effects of tea catechin Inhalation on methicillin-resistant Staphylococcus aureus in elderly patients in a hospital ward. J Hosp Infect. 2003;53:229–31 10.1053/jhin.2002.1327 12623326

[20] Hayashi K, Sato K, Fukamachi S, Nagamine T Oral care for elderly persons with oral lesion. Gunma Hokengaku Kiyo. 2002;23:89–94 (in Japanese)

[21] Shimamura T The effect of tea on various microorganisms. Rinsho Kensa. 1989;33:323–4 (in Japanese)

[22] Shimamura T The prevention of MRSA infections by tea extracts. Rinsho Kensa. 1992;36:904–5 (in Japanese)

[23] Yamada H, Takuma N, Daimon T, Hara Y Gargling with tea catechin extracts for the prevention of influenza infection in elderly nursing home residents: a prospective clinical study. J Altern Complement Med. 2006;12:669–72 10.1089/acm.2006.12.669 16970537

[24] Otsubo Y, Seki R, Akabane S, Uehara Y, Kondou S The potential of gargling with tea. Kangogaku Zasshi. 2000;64:778–81 (in Japanese)

[25] Cohen S Social status and susceptibility to respiratory infections. Ann N Y Acad Sci. 1999;896:246–53 10.1111/j.1749-6632.1999.tb08119.x 10681901

